# Generalised muscle weakness after bladder wall injection of Abobotulinum Toxin A: experience of a woman with tetraplegia who required increased caregiver support: importance of doctor–patient communication: duty of candour for spinal cord physician and responsibilities of a patient

**DOI:** 10.1038/s41394-018-0128-x

**Published:** 2018-11-13

**Authors:** Subramanian Vaidyanathan, Tracey Foster, Bakul M. Soni

**Affiliations:** 1 Regional Spinal Injuries Centre, Southport and Formby District General Hospital, Town Lane, Southport, PR8 6PN United Kingdom; 2 Lancashire, United Kingdom

## Abstract

**Introduction:**

Generalised muscle weakness can occur after bladder wall injection of Abobotulinum toxin and the patient may require additional caregiver support.

**Case presentation:**

A woman with C-8 AIS A tetraplegia received bladder wall injection of Abobotulinum toxin A 1000 units for detrusor over-activity. After 2.5 weeks, she developed weakness of arms; could not lift herself for pressure relief; could not transfer using sliding board; she regained the original muscle strength in 6 weeks. After 13 months, Abobotulinum toxin A 1000 units were injected into detrusor. Ten days after the second Abobotulinum toxin A injection, she developed generalised muscle weakness. She had not regained full function in her arms and hands 8 months later.

Prior to bladder wall injection of Abobotulinum toxin A, this patient was not aware that she could develop muscle weakness albeit very rarely. Therefore, the patient made no association of the muscle weakness, which occurred after the first injection, to Abobotulinum toxin A. For this reason, she did not inform the clinicians that she developed weakness of upper limbs following Abobotulinum toxin A injection. As she was not informed of this side effect before the second bladder wall injection of Abobotulinum toxin A, she consented to undergo the repeat procedure and developed generalised muscle weakness.

**Discussion:**

Patients should inform doctors the adverse effects of medical therapy so that future treatment is amended to ensure patient safety. Professional duty of candour states that doctors should discuss risks which occur often, those that are serious even if very unlikely, and those that are important to the patient.

## Introduction

Muscle weakness is an important complication after Abobotulinum toxin A injection in patients with neurogenic detrusor over-activity [1–10]. Generalised muscle weakness following injection of botulinum toxin in to detrusor may not be life threatening but can be very disabling to some spinal cord injury patients. A man with C8 AIS A tetraplegia, who received 300 units of botulinum neurotoxin A experienced general weakness of the arm muscles, which substantially influenced his transfers and he lost the qualification for the Paralympics [9]. The information sheet on botulinum toxin injections into the bladder wall, published by British Association of Urological Surgeons (BAUS), mentions generalised weakness of the legs and arms, which usually settles without admission or treatment; the consent form does not state explicitly potential impact of muscle weakness upon a person with tetraplegia and his/her care needs [11].

The aim of this case report is to raise awareness amongst health professionals and patients towards:

1. Generalised muscle weakness can occur after bladder wall injection of Abobotulinum toxin A in persons with spinal cord injury and additional caregiver support should be made available if required.
2. Spinal cord physicians should discuss the risks that occur often, those that are serious even if very unlikely, and those that the patient is likely to think are important in accordance with the professional duty of candour.
3. Similarly, patients should be advised to communicate all adverse effects of medical therapy back to the doctors so that future treatment can be amended to prevent recurrence of side effects and ensure patient safety.

## Case presentation

In 1995, a 25-year-old lady was on her way to work riding a bicycle when she collided with a van parked on the bicycle lane. In the hospital, X-ray of cervical spine revealed C-6/C-7 dislocation. Clinical examination revealed C-8 AIS A tetraplegia. She was treated nonoperatively. Following rehabilitation, this patient had been managing her bladder by intermittent catheterisations performed by caregivers and intra-vesical instillation of oxybutynin 5 mg solution four times a day.

In 2014, this patient developed recurring bladder spasms and urine leakages in between intermittent catheterisations. She could not retain oxybutynin intra-vesical instillations; the solution would come out as soon as it went inside the bladder. This patient experienced symptoms of autonomic dysreflexia including blotches on her legs, sharp pain in her head, hot feeling on her face and bladder pain with bladder spasms. Once caregivers performed catheterisation, blotches went away and headache was relieved; feeling of warm sensation disappeared, but bladder pain persisted. She was prescribed mirabegron 50 mg once a day from June 2014 to January 2015; the patient felt that mirabegron did not work as it made no difference to urine leakages. She was then prescribed oxybutynin by mouth and transdermal application of oxybutynin from January 2015. This patient had been taking oral baclofen since 1995; baclofen had no effect on bladder spasms although oral baclofen reduced spasms in her back and legs.

This patient found it necessary to catheterise every couple of hours. Despite such frequent catheterisations, she had urinary leakages between catheterisation, which affected her quality of life. She found it difficult to cope physically and mentally with this situation. The patient did not want to live with a permanent catheter and urine bag, she had used a permanent catheter for social occasions but sometimes she would bypass. This patient had come to terms that she would be unable to walk but she could not accept to live with permanent urinary catheter and leg bag.

Videourodynamics was performed in October 2014; initial residual urine was small. The filling phase showed gross detrusor over-activity. The reflex volume was less than 70 ml; pdet went up to 50 cm H_2_O with spontaneous emptying. While doing stress leakage, there was no stress leakage; the bladder neck was well supported. The second fill showed exactly the same findings in spite of a very slow fill.

The patient’s first referral to the Urology Clinic focused on:

- Eligibility to have the bladder wall botulinum toxin injection at the tertiary care hospital.
- Confirmation of the problems the patient was experiencing and that relevant urology tests and video urodynamic studies had been performed and were current.
- Potential risk of getting autonomic dysreflexia and what measures needed to be in place before any procedure was performed.
- The patient was told she met the criteria for the Bladder botulinum toxin injection and would be put on the waiting list.
- Discussion about the procedure of botulinum toxin injection to the bladder, and how it is performed under local anaesthesia using flexible cystoscopy.

There was no conversation about the dose of Abobotulinum toxin A the patient should receive. The patient received no verbal communication or written literature regarding possibility of generalised muscle weakness occurring after bladder wall injection of Abobotulinum toxin A and potential impact of muscle weakness on her care needs. Risks outlined to the patient at the first procedure were: possibility of urine infection, passing blood in urine and slight spotting. The risk of generalised muscle weakness after Abobotulinum toxin A injections was not explained to the patient at any time before or after the procedure.

Abobotulinum Toxin A 1000 units were injected into the urinary bladder under local anaesthesia in May 2016 in a nearby tertiary care hospital but it was performed in the operation theatre because of concerns about autonomic dysreflexia. On discharge, the patient was informed to contact her General Practitioner if she developed symptoms of urine infection. The patient did not receive any literature with regard to side effects of muscle weakness following bladder wall injection of Abobotulinum toxin A. About 2.5 weeks later, this patient noticed weakness of her arms. Muscle weakness did not happen abruptly; muscle weakness occurred gradually after 2.5 weeks following Abobotulinum toxin A injection. When she was lifted on to the platform for a bath, she could not lie down herself or lift herself up. She could not move forwards or backwards without support. After Abobotulinum toxin A injection, she could not lift herself for pressure relief. She could not transfer herself using a sliding board whereas she was doing transfers using a sliding board very easily prior to Abobotulinum toxin A injection. She could not put her arm in to her coat. She experienced worsened balance and felt unstable when she leaned forward. She did not develop difficulty in swallowing. In about 6 weeks, she regained her muscle strength.

Approximately 3 weeks after Abobotulinum toxin A injection, the patient had a close family bereavement, which had affected her emotionally. The patient’s muscle weakness having lasted for a short period of time and having no knowledge of the full side effects or risks of botulinum toxin, and her mind preoccupied by the recent bereavement, the patient made no causal association between the muscle weakness and the bladder wall injection of Abobotulinum toxin A even though the muscle weakness occurred soon after Abobotulinum toxin A therapy. During the follow-up appointment in October 2016, the length of time that had passed after the occurrence of muscle weakness also contributed to the failure to communicate this side effect back to the physician.

In October 2016, the patient was noted not to get any significant urgency or urinary leak, however, she started getting symptoms of urinary tract infections and she was prescribed nitrofurantoin 50 mg nocte. In February 2017 the symptoms of neurogenic over-activity had returned; flexible cystoscopy and intra-vesical injection of botulinum toxin were planned under local anaesthesia but for an Anaesthetist to be on standby in case this patient developed autonomic dysreflexia. Now in hindsight, the patient felt that had she been aware of the possibility of generalised muscle weakness after botulinum toxin injection, she would not have consented to the second bladder wall injection of Abobotulinum toxin A; again with the hindsight, she felt she would have requested for an immediate follow-up appointment with her consultant urologist.

In June 2017, Abobotulinum toxin A, 1000 units, was injected into the urinary bladder in the same hospital where the first botulinum toxin injection was administered. The consent form used for both procedures did not contain any warning regarding potential risk of distant spread of muscle weakness or a ‘Black Box Warning’ as stated by FDA. The black box warning states ‘Post-marketing safety data from approved botulinum toxins suggest that botulinum toxin effects may, in some cases, be observed beyond the site of local injection. The symptoms are consistent with the mechanism of action of botulinum toxin and may include asthenia, generalised muscle weakness, diplopia, ptosis, dysphagia, dysphonia, dysarthria, urinary incontinence and breathing difficulties. These symptoms have been reported hours to weeks after injection. Swallowing and breathing difficulties can be life threatening and there have been reports of death related to spread of toxin effects.’ [12]

Weakness of muscles came quicker after the second injection of Abobotulinum toxin A. Ten days after Abobotulinum toxin A injection, this patient could not sit up or lie down independently. Weakness of arms as well as the hands was noticeable; she was easily fatigued. Muscle weakness was severe in her arms, hands and trunk. When she tried to put her coat on, she did not have the strength to push her arm through the coat. She could not open a blister tablet pack, was unable to lift lightweight objects and she was struggling to hold a pen. Prior to Abobotulinum toxin A injection, she could lift herself so that her partner could apply soap and wash her buttocks. Following Abobotulinum toxin A injection, she could not lift herself and her partner was unable to clean her buttocks. The patient’s respiratory muscles were affected; she could not project her voice as strongly as she did prior to Abobotulinum toxin A injection. When she was sat up, she was struggling to call her caregivers. She was not able to shout for her partner across the road when they went out. She could not cough as effectively as she did before Abobotulinum toxin A injection. She had no truncal balance; she was very concerned that she might fall out of her wheel chair. The patient’s blood pressure was severely affected; blood pressure would become very low when first sitting up in the morning. Patient suffered with light-headedness and on one occasion, passed out. Prior to Abobotulinum toxin A injection, she only drank 300 ml of milk with morning medication when sat up. Now she needed caregiver support to sit up and required to drink an additional 500–750 ml of water before blood pressure would become stable.

In October 2017, this patient felt that she had not regained strength in her triceps fully. The weakness of her hands also persisted. She was still unable to lift herself after she was laid in the bath. When she was going down the drive in her wheelchair, she was losing control of her wheelchair. When she was holding a pen, she could not continue to grip the pen after writing one sentence. She could not apply enough pressure to write legibly. She was unable to lift herself and move to the left for car transfers. She could not position herself on the chair. She was still unable to do pressure lifts. She was no longer self-caring; everything was being done for her.

In February 2018, the patient had regained some muscle strength in her arms; however, daily exercise of biceps and triceps was still on going to facilitate possible improvement to the level which she had prior to Abobotulinum toxin A injection. Patient’s pincer grip and strength in both hands was still weak with slower recovery. Self-propelling of wheelchair was still difficult, particularly on uneven surfaces. She was not confident to push her wheelchair alone when outdoors. The patient felt it would take longer for her to regain dexterity and strength in her hands and fingers to the pre Abobotulinum toxin A injection status.

After Abobotulinum toxin A injection, this patient could not stop oxybutynin. She required to continue oxybutynin 5 mg twice a day by mouth and applied oxybutynin skin patch changing it every third day.

Following Abobotulinum toxin A injection, this patient noticed that she had to take an increased dose of senna to maintain regular bowel movements. Before Abobotulinum toxin A injection, she was taking 18 ml of senna; after Abobotulinum toxin A injection, this patient had to increase the dose of senna to 30 ml.

In the timeline from the first appointment in May 2016 to her final appointment in October 2017, the patient had been transferred from one consultant to another consultant, and had seen four different doctors. In October 2017, the physician in a tertiary care hospital advised the patient not to have any further injection of botuinum toxin, as was apparently clear that the patient developed upper limb weakness and respiratory problems following Abobotulinum toxin A injection on both occasions.

During subsequent visit to the spinal unit, this patient was advised to continue oxybutynin tablets, transdermal oxybutynin patches and regular intermittent catheterisations.

## Discussion

Generalised muscle weakness has been described after use of Abobotulinum toxin A for neurogenic detrusor over-activity. Our patient underwent bladder wall injection of 1000 units of Abobotulinum toxin A twice and developed generalised muscle weakness on both occasions. Published reports on the dose of Abobotulinum toxin A and occurrence of muscle weakness are given below (the list is not exhaustive).

- Ruffion et al. [13] stated that a dose of 1000 Speywood Units of Abobotulinum toxin A probably had a more prolonged effect than 500 SU but exposed the patient to major complications.
- A woman patient with tetraplegia, reported by Wyndaele and Van Dromme [9] received 1000 units of Abobotulinum toxin A, developed generalised muscle weakness for around 3 months, which caused her severe difficulties for transfers out of the wheelchair.
- Akbar et al. [14] reported three patients with spinal cord injury, who received a dose of 1000 units Abobotulinum toxin A and developed systemic side effects and generalised muscle weakness. These resolved without intervention and did not recur after reducing the adult dose to 750 units (paediatric dose 20 units/kg, not > 400 units), which seemed to be the optimum dose for good efficacy with an adequate safety margin.
- Grosse et al. [15] observed transient muscular weakness in the trunk and/or extremities in four out of 66 patients after Abobotulinum toxin A^®^ injections for neurogenic lower urinary tract dysfunction. Muscle weakness lasted for 2 months after 1000 UI/10 ml in one patient; for 4 weeks after 750 UI/10 ml in two patients (one of these patients had two previous Onabotulinum Toxin A injections 300 UI/15 ml without problems); the fourth patient suffered for 2 months after Abobotulinum toxin A 1000 UI/10 ml, for 6 weeks after Abobotulinum toxin A 750 UI/10 ml, and again for 6 weeks after Abobotulinum toxin A 750 UI/5 ml. These authors proposed that generalised muscular weakness was caused by systemic dispersion of Abobotulinum toxin A which in turn depended on the dosage and dilution volumes.

### Generalised muscle weakness: possible mechanism and relationship to dosage

In the existing literature on using Abobotulinum toxin A for neurogenic detrusor over-activity, the largest study was published by Del Popolo et al. [16]. These investigators found no statistically significant differences in efficacy or duration between the three Abobotulinum toxin A doses: 500, 750 and 1000 units; their study suggested that using 1000 units for a first dose might not be justified. It is unlikely that the Abobotulinum toxin A 1000 units (and not a lower dose) would have been administered during the repeat procedure in our patient if occurrence of muscle weakness had been discussed with the patient by the clinicians prior to Abobotulinum toxin A injections, which would have prompted the patient to inform the clinicians that she indeed developed transient muscle weakness after the first injection.

Crowner et al. [10] stated that risk for systemic effects is related to total injection dose and injection frequency. These authors postulated that distant spread might be mediated by direct entry of botulinum toxin into the vascular system through the capillary field or venous system, which might be influenced by the volume of fluid used for botlulinum toxin reconstitution. Capillary uptake is likely greater when using larger doses of toxin and/or larger volumes of fluid for injections. Rapp et al. [17] reviewed injection techniques and protocols for botulinum toxin; they found no case of generalised muscle weakness after using lower doses of both Onabotulinum Toxin A (200 U) and Abobotulinum toxin A (500 SU).

Our case illustrates the responsibility of both the doctor and spinal cord injury patients. The physician should make sure that the patient understood the benefits and risks of a procedure. The professional duty of candour [18] stipulates that a doctor must give the patient clear, accurate information about the risks of the proposed treatment and of any reasonable alternative options, and check that the patient understands. Such a discussion must include the risks that occur often, those that are serious even if very unlikely, and those that the patient is likely to think are important [19]. As much as it is desirable that the physician communicates with the patient as per the judgement of Supreme Court of United Kingdom [19], it is also very important for the patient to communicate all side effects of treatment to the physician.

In this case it appears that the patient did not know the serious but rare side effects of Abobotulinum toxin A injection e.g. generalised muscle weakness that could occur after botulinum toxin injection. Therefore, the patient made no connection that generalised muscle weakness, which occurred after the first injection, was probably related to Abobotulinum toxin A. Hence, she did not inform the clinicians of its occurrence; she proceeded to have the second injection and developed generalised muscle weakness once again.

## Learning points

1. Patients and caregivers should be made aware that the systemic effects can happen hours, days to weeks after an injection of botulinum toxin injection; they should seek medical advice without delay if they develop difficulty in swallowing or breathing.
2. If generalised muscle weakness occurs, the patient’s independence can be curtailed and the patient may require additional caregiver support. Possible need for increased caregiver support should be discussed with the patient before administering botulinum toxin injection into urinary bladder.
3. Professional duty of candour states that a doctor should discuss risks that occur often, those that are serious even if very unlikely, and those that the patient is likely to think are important.
4. Patients should be advised to communicate all adverse effects of medical therapy to doctors so that future treatment can be amended to prevent recurrence of side effects and ensure patient safety.
5. Patients should receive a specific information leaflet and consent form for botulinum toxin. For example, the patient leaflet from Addenbrooke’s Hospital, Cambridge, UK, states [20]: “Please use the check boxes to tick off individual items when you are happy that they have been discussed to your satisfaction” “Under Rare (less than 1 in 50):

Generalised weakness due to the effect of the toxin on the muscles of the body, requiring admission to hospital”
